# Selected Biomarkers of Depression: What Are the Effects of Cytokines and Inflammation?

**DOI:** 10.3390/ijms24010578

**Published:** 2022-12-29

**Authors:** Stefan Harsanyi, Ida Kupcova, Lubos Danisovic, Martin Klein

**Affiliations:** 1 Institute of Medical Biology, Genetics and Clinical Genetics, Faculty of Medicine, Comenius University in Bratislava, Sasinkova 4, 811 08 Bratislava, Slovakia; 2 Institute of Histology and Embryology, Faculty of Medicine, Comenius University in Bratislava, Sasinkova 4, 811 08 Bratislava, Slovakia

**Keywords:** depression, cytokines, interleukins, chemokines, lymphokines, IL-1β, IL-12, TNF

## Abstract

Depression is one of the leading mental illnesses worldwide and lowers the quality of life of many. According to WHO, about 5% of the worldwide population suffers from depression. Newer studies report a staggering global prevalence of 27.6%, and it is rising. Professionally, depression belonging to affective disorders is a psychiatric illness, and the category of major depressive disorder (MDD) comprises various diagnoses related to persistent and disruptive mood disorders. Due to this fact, it is imperative to find a way to assess depression quantitatively using a specific biomarker or a panel of biomarkers that would be able to reflect the patients’ state and the effects of therapy. Cytokines, hormones, oxidative stress markers, and neuropeptides are studied in association with depression. The latest research into inflammatory cytokines shows that their relationship with the etiology of depression is causative. There are stronger cytokine reactions to pathogens and stressors in depression. If combined with other predisposing factors, responses lead to prolonged inflammatory processes, prolonged dysregulation of various axes, stress, pain, mood changes, anxiety, and depression. This review focuses on the most recent data on cytokines as markers of depression concerning their roles in its pathogenesis, their possible use in diagnosis and management, their different levels in bodily fluids, and their similarities in animal studies. However, cytokines are not isolated from the pathophysiologic mechanisms of depression or other psychiatric disorders. Their effects are only a part of the whole pathway.

## 1. Introduction

Depression is one of the leading mental illnesses worldwide and lowers the quality of life of many. According to WHO, about 5% of the worldwide population suffers from depression. Some pessimistic scenarios estimate that by the end of 2030, it should become the leading cause of the disease burden. However, these predictions are based on data from 1990, not considering many recent problems such as wars, economic crises, and the coronavirus pandemic [1,2]. Newer studies report a staggering global prevalence of 27.6%, and while the prevalence is rising globally, the late SARS-CoV-2 pandemic caused problems, especially in middle- and low-income countries [3,4]. Professionally depression belonging to affective disorders is a psychiatric illness, and the category of major depressive disorder (MDD) comprises various diagnoses related to persistent and disruptive mood disorders [5]. It poses an issue for healthcare professionals, as in clinical practice, the variability in patient presentation, diagnosis, and management hinders their ability for treatment, and the prognosis often remains unpredictable [6]. Due to this fact, it is imperative to find a way to assess depression quantitatively using a specific biomarker or a panel of biomarkers that would be able to reflect the patients’ state and the effects of therapy. These markers can be obtained from serum, saliva, cerebrospinal fluid (CSF), urine, feces, or hair, using a non-invasive liquid biopsy [7]. Prolonged stress and anxiety also contribute to the prevalence of depression. However, the etiology of depression is multifactorial, affected by genetic predisposition, epigenetic modifications, and environmental and psychosocial factors, complicating the identification of these biomarkers.

Cytokines, hormones, oxidative stress markers, and neuropeptides have been studied in association with depression [8]. The dysregulation of the hypothalamus–pituitary–adrenal (HPA) or hypothalamus–pituitary–gonadal (HPG) axes, along with, e.g., depletion of brain serotonin or the effects of inflammation and the immune system response, are observed in depression [9,10]. Thorough research of the pathophysiological mechanisms has uncovered new pathways to which therapeutic interventions can be applied [11]. New treatment targets, be they psychological or pharmaceutic, are constantly being developed, as depression can often be therapy-resistant [12,13]. Preclinical and clinical studies associate oxidative stress with affective disorders (including depression) where antioxidants (e.g., vitamin E, zinc, glutathione, or coenzyme Q10) are the markers, and their impaired status can be corrected by a successful therapy [14]. Hormones and neuropeptides are usually associated with serotonergic, dopaminergic, noradrenergic, and GABA systems [6].

On the other hand, cytokines come with cell signaling, the immune system, and their response to internal and external factors such as inflammation. However, their peripheral levels are also studied in association with antidepressant treatment [15,16]. These small proteins comprise interleukins, interferons, chemokines, lymphokines, and tumor necrosis factors (TNFs or the tumor necrosis factor superfamily). Transforming growth factor beta (TGF-β) belongs to the transforming growth factor superfamily of cytokines that comprises TGF-β1, TGF-β2, and TGF-β3. The link between both acute and chronic inflammation is connected to depression, as the relationship between pro-inflammatory cytokines and depression has been studied for a long time [17]. Levels of cytokines have been found significantly altered in depression, and patients, even if otherwise clinically healthy, show elevated or decreased levels of various cytokines and their soluble receptors in bodily fluids [18]. There is a disparity in immune suppression and activation in depression; this is a cycle of cause/effect and leads to impaired cellular immunity and inflammation [19]. There are stronger cytokine reactions to pathogens and stressors in depression, in which the pro-inflammatory response induces an anti-inflammatory answer. These findings complement the theory of two opposite, counter-regulating systems: the immune-inflammatory response system (IRS) and the compensatory immune-regulatory reflex system (CIRS) [20]. If combined with other predisposing factors, these responses lead to a prolonged fight of pro-inflammatory and anti-inflammatory processes, prolonged dysregulation of various axes, stress, pain, mood changes, anxiety, and ultimately, depression [21,22].

This review focuses on the most recent data on cytokines as markers of depression concerning their roles in its pathogenesis, their possible use in diagnosis and management, their different levels in bodily fluids, and their similarities in animal studies. However, cytokines are not isolated from the pathophysiologic mechanisms of depression or other psychiatric disorders. Their effects are only a part of the whole pathway. Only for this review are these cytokines taken into consideration one by one to better show their individual effects and relations in this much broader system. This review article will refer to depression or MDD, depending on the source.

## 2. Cytokines

**Figure 1:**
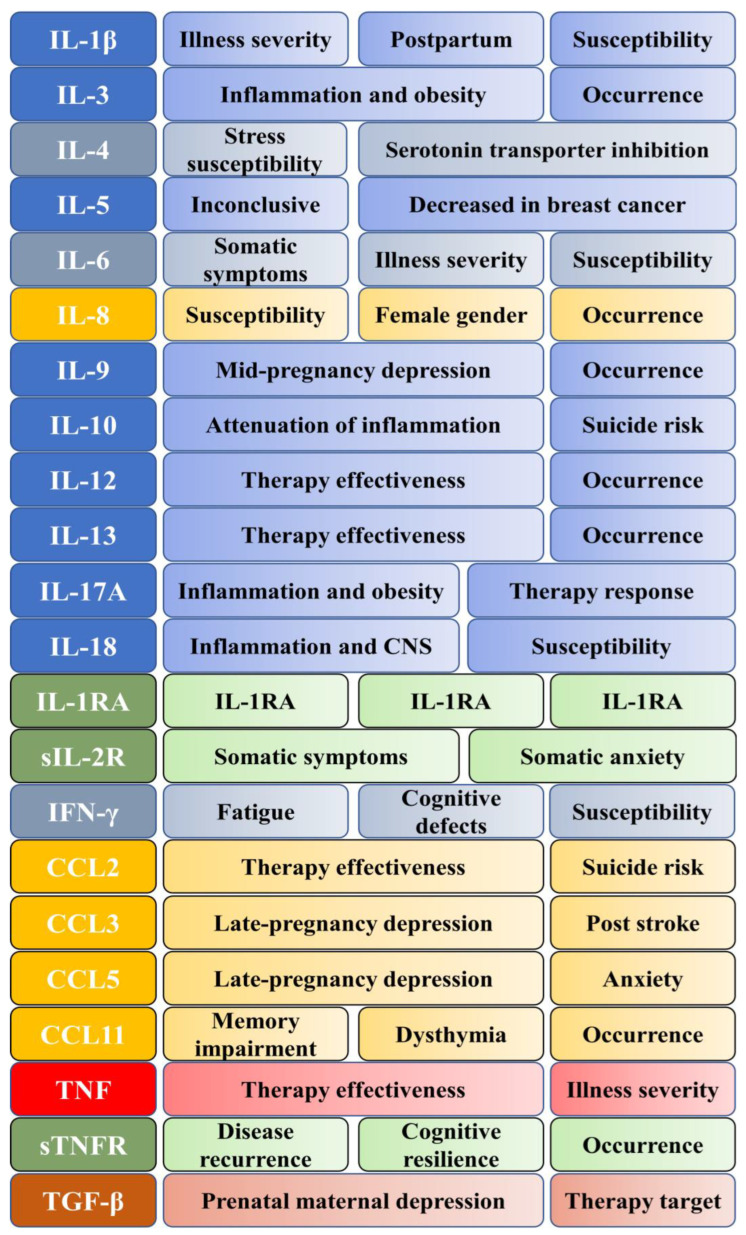
Cytokines and their main associations with depression. Blue—interleukins, gray—lymphokines, yellow—chemokines, green—soluble receptors, red—tumor necrosis factor, brown—transforming growth factor.

Cytokines are a group of small heterogenous peptides that affect inflammation, cell proliferation, and differentiation. Cytokines have been heavily discussed in stress, anxiety, and depression [23]. Various cytokines released in the body are transported in the blood to effector cells, in the case of depression, to the brain, where they affect the neurotransmitter systems [24,25]. In Table 1, we present currently studied cytokines categorized into groups along with receptors and ligands. The effects of interleukin-1 beta (IL-1β), IL-1 receptor antagonist, interleukin-6 (IL-6), tumor necrosis factor-alpha (TNF-α), or interferon-gamma (IFN-γ) have been studied for a long time [26,27,28,29]. In recent years, more cytokines, their receptors, and ligands have been studied in association with mood worsening, depression risk, and depression [30]. T helper 17 cells (Th17) with IL-17A are studied in association with neuroinflammation [31]. Meta-analyses and systematic reviews show that levels of IL-1β, IL-2, IL-4, IL-8, the soluble IL-6 receptor (sIL-6R), IL-5, CCL3, IL-17A, and TGF-β1 are significantly changed in depression [16,32,33,34,35]. Figure 1 depicts all major cytokines mentioned in this article, their links, and their contributions to various kinds of depression. 

### 2.1. Interleukin-1 Beta

Interleukin-1 beta (IL-1β) is a pro-inflammatory cytokine encoded by the *IL1B* gene, located at 2q14.1. This cytokine was first implicated in the etiology of sickness behavior and later linked to depressive disorder along with IL-6 and TNF [36,37]. Elevated levels of various inflammatory markers have been found in episodes of depressive behavior. However, the elevation of pro-inflammatory cytokines is primarily a reaction to acute or chronic stress, where IL-1β was found to be elevated in 38.5% and 75.6% of studies, respectively [38,39]. Moreover, danger signals are one of the inducers of the inflammatory response. This happens via inflammasomes or toll-like receptors (TLRs) and leads to elevated IL-1β, IL-6, and TNF-α [40,41]. Untreated patients suffering from depression have been found to have elevated IL-1β levels in CSF. This raises the question of whether this occurrence is more related to depression or acute reaction to stress, as mice under stress expressed more IL-1β than controls [42,43]. This association was later studied by Thomas et al., who found that elderly subjects (above 60 years) expressed IL-1β proportionally to illness severity [44]. Corwin et al. reported elevated IL-1β levels in women suffering from postpartum depression (PPD) [45]. A meta-analysis concluded that IL-1β was significantly elevated in patients with depression and Alzheimer’s disease (AD), while IL-6 was elevated only in patients with depression [38]. Recently, Ferreira et al. reported a possible association of SNP in the *IL1B* gene (rs16944; 511 C > T) with higher susceptibility to depression in patients with multiple sclerosis (MS) [46].

Ovaskainen et al. studied IL-1β in males and concluded that decreased levels of this cytokine and elevated levels of the interleukin-1 receptor antagonist (IL-1RA) were more frequent in depressed subjects [47]. These findings contradict each other even if there is a sex difference. Noteworthy, however, is the relation between IL-1β and the P2X family of receptors, in which P2X7 inhibition has been associated with antidepressant-like effects, and IL-1β knockout mice failed to express these receptors [48,49]. Stress-induced production of pro-inflammatory cytokines was reported by Portfield et al., where repeated stressor exposure in rats enhanced the production of IL-1β via the norepinephrine beta-adrenergic pathway [50]. However, a subsequent study by Barnard et al. reported only male rats to be affected by the beta-adrenergic pathway, while females remained unaffected [51]. The use of intrahippocampal IL-1RA causes downregulation of brain-derived neurotrophic factor (BDNF) in individuals experiencing social isolation [52]. IL-1RA modulated synaptic plasticity in a rat AD model, where acute beta-amyloid mediated potentiation impairment was possibly caused by inflammatory response and release of IL-1β [53]. IL-1Ra knockout mice show anxiety-like behavior as they grow older [54]. Receptor upregulation has been found in patients suffering from migraine with aura symptoms, believed to be associated with cortical spreading depression [55]. This upregulation may be a response to modulate the inflammatory process.

### 2.2. Soluble Interleukin-2 Receptor

The soluble IL-2 receptor (sIL-2R) is approx. 40 kDa in size and results from proteolytic cleavage of the ectodomains of the membrane-bound IL-2Rα chain as a result of T cell activation. Its levels are increased in inflammation, infections, and autoimmune disease. sIL-2R has a low affinity for IL-2 and can function either as a decoy receptor reducing the bio-availability of IL-2 or by enabling trans presentation to cells expressing the dimeric, intermediate-affinity IL-2R, which leads to enhanced or suppressed immune response depending on the type of cells involved [56,57]. The role sIL-2R may play in depression was first mentioned in the literature in 1991 in an article exploring depression-related disturbances in mitogen-induced lymphocyte responses together with the production of IL-1β and sIL-2R, suggesting that T cell activation may be one of the characteristics of MDD [58]. Serum levels of sIL-2R are increased in MDD and correlate with somatoform symptoms and somatic anxiety in depression but not with the overall severity of the disease [56,59]. That serum sIL-2R was increased in MDD was later reaffirmed, with the mean difference in sIL-2R levels between healthy subjects and MDD patients being 0.555 U/mL [60].

### 2.3. Interleukin-3

Interleukin-3 (IL-3) is a growth-promoting cytokine encoded by the *IL3* gene located at 5q31.1 [61]. It is a multipotent hematopoietic growth factor expressed mainly by activated T-lymphocytes and macrophages inducing proliferation, maturation, and perhaps self-renewal of stem cells committed to the hematopoietic lineage [62]. The relationship between serum IL-3 and psychiatric conditions was reported by Xiu et al., who focused on schizophrenic patients. Compared to healthy controls, the study group had significantly elevated IL-3 levels. Moreover, IL-3 levels were positively correlated with depressive sub-scores [63]. The most recent 2022 paper by Gao et al. compared serum cytokine levels, including IL-3, in normal and overweight patients with their first depressive episode without the medication history of taking antidepressants. Of the 37 cytokines measured, IL-3 was significantly elevated in overweight, depressed patients compared to their normal-weight depressed counterparts [64]. These results indicate that these two inflammation-related conditions might share some aspects of their pathogenesis. A 2020 meta-analysis by Osimo et al. analyzed 107 studies comprising 5166 patients and 5083 controls and found IL-3 among those cytokines whose blood levels were significantly elevated in depressed subjects [65]. Overall, IL-3 seems to be another valuable marker of depression, despite there not being much information about its precise action.

### 2.4. Interleukin-4

Interleukin-4 (IL-4) is coded by the *IL4* gene, a 160 kb region on the long arm of chromosome 5. IL-4 plays a role in the regulation of IgE synthesis, differentiation of naive T cells into the Th2 subtype, suppresses IL-2 production, and promotes the expression of anti-inflammatory cytokines such as TGF-β1. IL-4 is produced by CD4 T cells, basophils, eosinophils, mastocytes, NK cells, and innate lymphoid cells type 2, while production in CNS by microglia and neuronal cells [66,67,68]. Its effect on the brain is of particular importance for microglia specialization. Microglia exposed to IL-4 and IL-13 specialize to the M2 anti-inflammatory subtype in contrast to the pro-inflammatory M1 subtype induced by exposure to IFN-α, TNF-β, or lipopolysaccharide (LPS). IL-4-driven Arg1^+^ microglia in the hippocampus trigger brain-derived neurotrophic factor (BDNF)-driven neurogenesis in reaction to stress, resulting in improved resilience to stress-induced depression. In a murine model, mice were exposed to chronic mild stress, and subsequently, levels of IL-4 and other cytokines in their hippocampi were measured using an immunofluorescence assay. The results showed overexpression of IL-4 in the mice group with low-stress susceptibility [69,70]. IL-4 inhibits the serotonin transporter activity in a dose-dependent manner, hence increasing the amount of serotonin available, acting similarly to some of the currently used antidepressants. This is supported by the fact that the use of an anti-IL-4 antibody reversed the effect IL-4 had on serotonin transporter levels in an experiment using B lymphoblasts. In a later animal model, rodents treated with IFN-α showed more depressive behaviors in the forced swim test, tail suspension test, and active avoidance task when their IL-4 microglial reactivity was reduced [70,71]. In agreement with these findings, IL-4−/− mice tested in the forced swim test and tail suspension test showed significant depression-like behaviors compared to wild-type rodents, which was not further increased by the application of IFN-α [72].

### 2.5. Interleukin-5

Interleukin-5 (IL-5) is coded by a gene clustered with IL-3 and IL-4 on chromosome 5. It is involved in the activation, proliferation, and differentiation of eosinophils and is best known for its involvement in the pathophysiology of autoimmune disorders such as asthma and Graves’ disease [73,74]. IL-5 is produced by activated Th2 cells, microglia, mastocytes, and astrocytes; has mitogenic effects on microglia; and activates JAK2 and STAT5 pathways involved in cell proliferation, which can lead to depressive symptoms via glucocorticoid signaling. IL-5 is also involved in Ras-ERK pathways, the hyperactivity of which causes deficits in synaptic plasticity and hippocampal-related learning in mice [74,75,76]. Although the role of IL-5 in autoimmune and parasitic diseases is well described, little is known about its potential involvement in depressive disorder, and the findings have been inconsistent so far. Elomaa et al. described elevated IL-5 levels in 58 patients with MDD, though a later meta-analysis did find changes in levels of IL-5 to be inconclusive in depressed patients. Recent research on circulating cytokine levels in breast cancer patients even found IL-5 levels decreased in the depressed group [35,77].

### 2.6. Interleukin-6

The gene encoding interleukin-6 (IL-6) is located on chromosome 7, containing five exons [78]. IL-6 is released by Th1 and Th2 cells, having either pro- or anti-inflammatory properties, depending on the presence of either the IL-6 receptor or the membrane-bound gp130 signal transducer, which are expressed within specific cell types throughout the body. Microglia secrete IL-6 by interactions of SDF-1 with CXCR4, leading to enhanced IL-6 mRNA expression and increased protein synthesis by activating the MAPK/ERK signal pathway and phosphatidylinositol-3 kinase. Within the peripheral and central nervous systems, IL-6 can act as a neuronal growth factor leading to neurite development and nerve regeneration. In reaction to stressful stimuli, the synthesis of IL-6 increases, and findings suggest that in depression, higher IL-6 levels correlate with a more severe course of the disease [79,80,81]. It also seems to be linked to specific symptoms or subtypes of depressive disorder, as the relationship between IL-6 levels and reduced appetite, sleep disturbances, low mood, and feelings of worthlessness has been confirmed [82]. Recently, Li et al., studied peripheral IL-6 levels and changes in white matter for two years after stressful life events (SLEs) in 185 subjects using diffusion tensor imaging to elucidate its role in developing depression after SLEs. The participants exhibited high levels of IL-6 during the stress period and a corresponding decline in IL-6 levels as the stress period ended. IL-6 correlated with the overall level of stress. After the period of acute stress, the IL-6 white matter network differences were shown to be strongly associated with the interindividual variation in susceptibility to depressive disorder in healthy individuals with SLEs [80]. However, it is not yet clear if the elevated plasma levels of IL-6 also translate to the same changes within the brain and if the elevated IL-6 level is a cause or consequence of depressive symptoms, as the results regarding the correlation between plasma and CSF IL-6 levels vary. In animal models of depression, IL-6 was reduced in the CA1 region of the hippocampus of rats exhibiting depressive behaviors and IL-6 knockdown facilitated development of depressive behaviors. At the same time, overexpression of IL-6 led to a reduction in these behaviors [83,84]. These conflicting findings could mean that there is a more complex relationship between plasmatic and CSF levels of IL-6, that IL-6 is released after stressors in a neuroprotective fashion, or they could point to a different role of IL-6 in rodent models of depression compared to human patients with depressive disorder.

Soluble IL-6 receptor (sIL-6R) results from alternative mRNA splicing, proteolysis of the membrane-bound IL-6R, and the release of extracellular vesicles as it is involved in the IL-6 pro-inflammatory trans-signaling. It is the only IL-6 receptor type in astrocytes and neurons [85,86]. In addition to increased IL-6 levels, sIL-R6 levels were reported in depressed patients, with elevated levels of both persisting even after the start of antidepressant treatment [87,88]. Contrary to these findings, sIL-6R was decreased in the CSF of elderly depressed patients medicated with antidepressants in combination with neuroleptics or benzodiazepines [89]. The reasons for these inconsistencies are yet to be further explored.

### 2.7. Interleukin-8

Interleukin-8 (IL-8 or CXCL8) is a chemokine produced by, e.g., macrophages, microglia, or endothelial cells. The *CXCL8* gene codes IL-8 with a cytogenetic location of 4q13.3. Microglial IL-8 reacts to pro-inflammatory stimuli, and elevation in the periphery has been linked to schizophrenia, bipolar disorder, or autism spectrum disorders. At the same time, its connection to MDD is relatively new and inconsistent [90]. Inconsistencies start with sex differences in rats, where the IL-8 effect on disease severity was associated only with females [91]. Female rats with lower IL-8 levels are more susceptible to medication and electroconvulsive therapy (ECT) [92,93]. In human studies, breast cancer survivors (100% female) with higher basal IL-8 levels at the start of the study were less likely to experience recurrent depression [94]. However, inconsistencies continue, as in a study on geriatric depression (patients 70–84 years of age), higher levels of IL-8 and IL-6 in CSF were associated with current depression [95]. Elevated levels of IL-8 in CSF were also observed by Kuzior et al. in patients suffering from unipolar depression [96]. Whole blood sample examination in a randomized study reported the possibility of IL-8 effect on depression to be rather mitigative than enhancing, where patients with higher levels of IL-8 are less likely to suffer from inflammation-associated depression [97]. Partly in line with previous studies is a study on IL-8 in delirium, dementia, and depression by Sajjad et al., who found an increased IL-8 concentration in patients with delirium/depression. In contrast, the group with delirium/dementia expressed lower levels of IL-8 [98]. Suppose we try to put this disparateness in order. In that case, we can say that low IL-8 levels and susceptibility to treatment do not negate current depression in individuals with higher levels, as there is a possibility of elevated levels in the initial phases. Additionally, peripheral levels do not have to correlate with CSF, but to be sure on this matter, more data are needed to understand all the complications in this pathway.

### 2.8. Interleukin-9

The gene for interleukin-9 (IL-9) is located on chromosome 5 within the TH2 cytokine cluster in the region q31–35, consisting of five exons and four introns. Though initially believed to be a Th2 cytokine, it was recently revealed that it is copiously secreted by newly discovered Th9 cells. The binding of IL-9 with its receptor promotes cross phosphorylation of Janus kinase (JAK) 1 and JAK3, leading to the activation of signal transducer and activator of transcription (STAT) 1, 3, and 5 [99,100]. IL-9 has also been implicated in the pathophysiology of depression. Upregulation of the *IL9* gene was found in the post-mortem analysis of the brains of patients with MDD in the Brodman Area 10—a part or brain involved in the mediation of reward-related behavior. Elevated IL-9 levels were found to correlate with maternal mid-pregnancy symptoms of depression and anxiety [101,102]. Varshney et al., found elevated blood IL-9 levels in depressed patients with type 2 diabetes compared to diabetic patients without depressive symptoms [103]. Elevated IL-9 was also reported in the saliva of MDD patients. However, it is not yet thoroughly researched to what degree the changes in saliva correlate with changes in blood samples [33].

### 2.9. Interleukin-10

Interleukin-10 is coded by the *IL10* gene located on chromosome 1 (1q31–32) and contains five exons. By acting on dendritic cells (DCs) and macrophages, IL-10 inhibits the development of TH1- and TH2-type responses, acting as an anti-inflammatory cytokine [104,105]. IL-10 knockout mice showed learned helplessness in animal depression models, a symptom that could be reverted by application of IL-10, whereas IL-10 administration led to increased motor activity in wild-type mice. Decreased levels of IL-10 were described in humans with symptoms of depression, anxiety, and increased suicide risk in a population study, as well as in stroke patients who developed the depressive disorder. Yet the literature also yields studies that report increased IL-10 levels in MDD. One suggested explanation for this discrepancy is that IL-10 levels increase initially in response to acute inflammation connected with depression as a part of the compensatory immune system. However, if the inflammation is not successfully attenuated with a longer disease duration, the IL-10 levels eventually decrease [106,107,108,109]. This theory is also supported by the fact that several types of antidepressants increase IL-10 levels [110,111].

### 2.10. Interleukin-12

Interleukin-12 (IL-12) is a heterodimeric pro-inflammatory cytokine encoded by two genes for each subunit, *IL12A* and *IL12B*, located at 3q25.33 and 5q33.3, respectively [112,113]. It was first discovered and described in the 1980s as a result of experiments with cell culture of Epstein-Barr virus-transformed lymphoblastoid cell lines. The heterodimeric biologically active form is also referred to as IL-12p70 [114]. It is synthesized mainly by antigen-presenting cells (APCs) such as macrophages or DCs. Its primary function is to help combat intracellular infectious agents, and it can be mobilized within minutes [115]. It is a highly potent inducer of Th1 response in humans [116]. The potential role of IL-12/IL-12p70 (from now on, it will be referred to as just IL-12) in autoimmunity and its relation to the etiopathogenesis of neurodevelopmental disorders such as autism was first studied 26 years ago. Singh performed a study on randomly selected 20 autistic children. The study group had significantly higher plasma levels of IL-12 and IFN-γ [117]. They scrutinized the correlation and possible causation between plasma level of IL-12 and neuropsychiatric disorders, including MDD, bipolar disorder, and schizophrenia. Kim et al., published a comparative study recruiting 102 psychiatric patients, 34 of whom had MDD. The two principal findings were that, first, the plasma levels of IL-12 were significantly higher in patients with depression compared to controls, and second, there was a significant decrease in IL-12 plasma levels after eight weeks of antidepressant pharmacotherapy [118]. Lee and Kim reproduced similar results in a clinical trial [119]. Along the same lines, Sutcigil et al. found elevated serum levels of IL-12, which decreased after eight weeks of sertraline therapy [120]. A recent 2019 study presented a similar picture. Nowak et al. found that patients with MDD without any comorbidities had higher plasma levels of IL-12, which can be mitigated by a sub-anesthetic dose of ketamine which acts through the promotion of M2 phenotype in macrophages [121]. El-Tantawy et al. chose an original approach that investigated serum levels of a cytokine palette, including IL-12, in patients diagnosed with rheumatoid arthritis (RA). The main aim was to correlate the cytokine levels with psychiatric disorders, mainly depressive disorder, and anxiety, which are more common in RA patients. Again, the levels of IL-12 were substantially elevated in the study group [122]. Schmidt et al. compared the serum levels of nine different cytokines, including IL-12. They originally designed the study so that the cytokine levels in depressive disorder were put into perspective with other immune system-associated conditions, such as obesity. Unsurprisingly, IL-12 was elevated in depressed patients. Comparing the obese and non-obese subgroups, both had IL-12 significantly elevated. However, from the whole spectrum of studied pro-inflammatory cytokines, more were elevated in the non-obese group. However surprising it might seem at first glance, it can point to the fact that obesity causes the elevation of pro-inflammatory status by itself, independent of depression. Thus, in the case of depression, only a few cytokines are elevated compared to the baseline chronic pro-inflammatory status. Therefore, obesity can mask depression-induced cytokine alteration, making the cytokine level results harder to interpret. Strikingly, the authors also found that IL-12 was elevated in the unemployed and retired [123]. The effect of serotonin re-uptake inhibitor (SSRI) treatment in patients with generalized anxiety disorder and its influence on IL-12 and other cytokine levels was the principal focus of the 2019 study by Hou et al., who reported a significant reduction in anxiety and pro-inflammatory cytokines levels compared to baseline figures [124]. All these results have been corroborated by a 2017 meta-analysis which included 82 studies, underlining the reproducibility of IL-12 plasma/serum level elevation in depressed patients [35]. On the other hand, a 2020 systematic review and meta-analysis investigated whether the examination of baseline levels of peripheral cytokines can predict the responsiveness to the antidepressant treatment. There was no difference in IL-12 (plus ten other cytokines) levels between subsequent responders and non-responders [16]. Taken together, the investigation of IL-12 serum/plasma level can be clinically valuable within a complex diagnostic approach. However, it always has to be interpreted with caution since many other conditions, e.g., obesity, can alter the same pro-inflammatory pathways, overlapping with each other.

### 2.11. Interleukin-13

Interleukin-13 (IL-13), first discovered in 1993, is a cytokine encoded by the *IL13* gene at located 5q31.1 [125]. It is closely associated with the *IL4* gene (5q23-31), and the two gene products (IL-13 and IL-4) also share some functional characteristics [126]. IL-13 is generally defined as an anti-inflammatory cytokine. For instance, it can mitigate the pro-inflammatory activation of microglia in the brain, providing neuroprotection during ischemic stroke. However, its effects can be pleiotropic, so under certain circumstances, IL-13 can contribute to or fail to suppress pro-inflammatory responses [127,128,129]. Corren reviewed that IL-13 plays a central role in various pathogenetic aspects of allergic conditions, such as allergic asthma, including the B lymphocyte switch to IgG production [130]. Thus, it is primarily associated with Th2-mediated immune functions [131]. Discussing the role of IL-13 in psychiatric conditions, Pavón et al. found elevated IL-13 serum levels in MDD patients. The most probable explanation of this finding was that chronic MDD-associated stress causes the chronic elevation of cortisol, which can raise IL-13 in peripheral blood [132]. The relationship between neuroendocrine-immune regulation and stress is complex, including Th1 and Th2 responses, depending on the stress duration. While acute stress is Th1-mediated and can be beneficial, chronic stress elicits deleterious Th2 responses, which may disrupt immune system integrity [133]. Hernandéz et al. authored a controlled clinical trial focused on the effect of SSRI treatment on various cytokine levels in MDD patients. At week 0, the depressed subjects had significantly elevated serum levels of IL-13, with a staggering 69% reduction at week 52 [134]. Wong et al., reported conflicting results of decreased levels of IL-13 in MDD patients. They discussed that such discrepancy might have arisen due to differences in gender, age, or severity of the disorder [135]. Czysz et al., compared 19 inflammatory markers as potential MDD treatment prediction modeling tools. Only IL-13 was found to be able to predict differential treatment outcomes [136]. The above-cited meta-analysis by Köhler et al. underpins the association between peripheral IL-13 elevation and MDD [14]. Despite acting on the opposite side of the Th1/Th2 dichotomy, IL-13 is associated with MDD similarly to IL-12. In a recent 2021 study, Vai et al. attempted to investigate the relationship between inflammatory profile and suicide and found that plasmatic IL-13 was elevated in patients with a history of suicidal behavior [137]. The link between IL-13 and suicidal behavior was also presented by Tonelli et al., who examined the samples of orbitofrontal cortices, namely the Brodmann area 11 of suicide victims. One of the selection criteria for the particular cytokine panel investigated, which included IL-13, was the previously published association between allergies and depression or even suicide [138,139]. The investigation revealed an increased expression of IL-13 in this area, typically associated with decision-making and planning [140].

### 2.12. Interleukin-17A

Interleukin-17A (IL-17A) is a pro-inflammatory cytokine encoded by the *IL17A* gene located at 6p12.2 [141]. A member of the highly evolutionarily conserved IL-17 cytokine superfamily, it was first discovered in 1993 [142]. Later, IL-17A was revealed to be the signature cytokine of a specific population of CD4^+^ T cells, known as T helper 17 or Th17 cells. Over the almost 30 years after its initial cloning, many research teams found it to be more than just a pro-inflammatory cytokine. McGeachy et al. reviewed its physiological functions, which include wound healing, extracellular matrix remodeling, role in thermogenesis, and others. From the pathological perspective, IL-17A has been implicated in the pathogenesis of autoimmune diseases, obesity-associated chronic low-grade inflammation, tumorigenesis, neurodegeneration, depression, and autism [143]. Matusevicius et al. found that blood and CSF from patients with MS contained, compared to controls, higher numbers of IL-17A-expressing mononuclear leucocytes [144]. In 2021, Nothdurfer et al. observed that MDD non-responders to antidepressant therapy had significantly elevated IL-17A levels, supporting its clinical relevance as a marker of therapy resistance [145]. Medina-Rodriguez et al. analyzed stool samples taken from mice to investigate the potential relation between dysbiosis of the gut microbiome and MDD. They found a relationship between certain types of gut microorganisms and the development of depression-like symptoms. The primary cells which mediated these processes were IL-17A-producing Th17 cells [146]. Alvarez-Mon et al. enrolled 30 MDD patients and 30 control subjects and compared the Th1, Th2, and Th17 subsets of CD4^+^ T cells. The study data showed that the circulating Th17 cell count was higher with corresponding increased serum levels of IL-17-A. The IL-17A overexpression was observed in various activation/differentiation stages of CD4^+^ T cells, namely the naïve, central memory, and effector memory T cells [147]. Min et al. studied the role of IL-17A in developing PPD and found a link between the elevated peripheral levels of IL-17A and the risk of PPD development [148]. The bottom line is that IL-17A positively correlates with MDD and other related conditions and can be used in diagnostic practice and therapy success prediction.

### 2.13. Interleukin-18

Interleukin-18 is a pro-inflammatory cytokine encoded by the *IL18* gene located at 11q23.1. Belonging to the IL-1 superfamily, IL-18 can stimulate both the Th1 and Th2 lines of differentiation [149]. Its discovery dates back to 1989, when Nakamura et al., described an “unidentified” factor capable of stimulating IFN-γ production, only later on designated as IL-18, or based on its first known action, as an interferon-gamma inducing factor [150]. In 2005, IL-18 was already established as a key player in neuroinflammation and neurodegeneration, as reviewed by Felderhoff-Mueser et al. [151]. IL-18 (along with IL-1β) is associated with inflammasomes—cytosolic multiprotein complexes, which, upon stimulation, induce the synthesis of these two pro-inflammatory cytokines by the enzymatic action of caspases. Inflammasome activation can also lead to different types of cell death, including apoptosis and pyroptosis [152]. Studying the inflammasome pathways and their supposed relation to MDD, Wong et al. focused on genetically and pharmacologically induced inhibition of caspase-1 and found that both genetic suppression and minocycline-induced antagonism of this inflammasome-associated enzyme ameliorated depressive and anxiety-like behavior in mice. They also found that minocycline had a beneficial effect on the gut microbiome, hypothesizing about the presumed interconnection known as the microbiota–inflammasome–brain axis [153]. In 2020, Song et al. established a mice model of chronic stress to investigate the potential role of NLRP1 inflammasome in the pathogenesis of MDD. The results showed that chronic stress activated NLRP1 inflammasome in the hippocampus, which in turn induced the release of pro-inflammatory cytokines, including IL-18. The mice with knocked down NLRP1 inflammasome activity displayed diminished chronic stress-induced depressive-like demeanor, indicating that inflammasome-mediated IL-18-induced CNS inflammation can indeed lead to depression [154]. Interestingly, Fan et al., examined the serum levels of three cytokines, one of which was IL-18, but found IL-18 to be lower in depressed subjects. They hypothesized that the results might reflect that the patients were treated with antidepressants, which could have lowered the serum levels of IL-18 [155]. Controlling for this variable, Kokai et al. measured the serum levels of IL-18 in 13 patients. Eight were diagnosed with MDD and five with panic disorder, but none were taking any psychotropic medications. In this scenario, IL-18 serum levels were significantly elevated [156]. Alcocer-Goméz et al. directly compared the differences in IL-18 levels between 20 “no treatment” subjects and 20 treated with amitriptyline, examining NLRP3 inflammasome and caspase-1 activity. Following the previously cited studies, the expression of NLRP3 and caspase-1-associated genes was increased, correlating with the elevation of IL-18 serum levels [157]. Prossin et al. implemented an innovative approach and correlated the IL-18 plasma levels with positron emission tomography (PET)-visualized µ-opioid receptor activity using the selective radiotracer [11C] carfentanil. Unsurprisingly, baseline IL-18 values were higher in MDD subjects, but the most exciting finding was that the IL-18 rose after sadness induction in healthy subjects, correlating with µ-opioid receptor activity [158]. All in all, IL-18 serum/plasma levels seem to correlate with IL-18 action as a potent activator of MDD-associated inflammatory changes in the CNS, which can be used diagnostically.

### 2.14. Interferon-Gamma

Interferon-gamma (IFN-γ) is encoded by the *IFNG* gene located at 12q15. IFN-γ plays a role in stress-induced immune dysregulation [159]. Women with higher subjective stress after breast cancer surgery but before adjuvant therapy were found to have lower basal and IFN-γ-augmented NK cells and reduced T cell proliferative response to mitogens [160,161]. Additionally, antidepressants were found to suppress the release of IFN-γ by activated T cells [162]. Elevated production of IFN-γ by CD3^+^ CD4^+^ T lymphocytes was observed in fatigued and depressed patients suffering from MS compared to non-fatigued MS patients [163]. The effect of IFN-γ on mood abnormalities via the cannabinoid CB1 receptor pathway has been observed in mice [164]. A meta-analysis on cytokines in MDD reported measurements extracted from research articles including 131 depressed and 107 nondepressed subjects, in which concentrations of IFN-γ did not differ between these groups [165]. However, a different study observed significantly decreased serum IFN-γ levels in patients with MDD compared to healthy controls [166]. While decreased in base levels, the reactivity to mitogens in depressive patients is significantly enhanced [167,168]. A different take on IFN-γ by Zhang et al. studied microglia isolated from the hippocampus of IFN-γ-injected mice, where the proliferation of neural stem/precursor cells (NSPCs) was suppressed by the inhibition of the JAK/STAT1 pathway [169]. The IFN-γ injection led to impairment of adult hippocampal neurogenesis and led to cognitive defects and depression-like behavior [169]. IFN-γ signaling was reported to play a role in myelin disruption in patients with migraine with aura and depression [170]. Moreover, a genetic variant (+874) T/A) has been proposed as a possible risk factor for patients, as they could be predisposed to IFN-induced depression [171]. A recent review of in vitro and ex vivo studies on the effects of different classes of antidepressant drugs reported that many of these drugs prevent IFN-γ microglial activation [172]. The effect of *IFN-γ* polymorphism on tryptophan metabolism and therapy was studied by Myint et al., who reported that the presence of the IFN-γ CA-repeat allele 2 (homozygous) resulted in significantly lower serum tryptophan and 5-hydroxy indole acetic acid (5HIAA). In contrast, serum kynurenine was considerably higher [173]. These findings point to a strong link between IFN-γ and depression.

### 2.15. CCL2

The chemokine (C-C motif) ligand 2 (CCL2) is a member of a chemokine superfamily characterized by the N-terminal cysteine arrangement variability comprising four subfamilies. The CC chemokine subfamily is represented by those chemokines which contain two adjacent cysteine residues, hence the name. CCL2 is encoded by the *CCL2* gene located at 17q12, along with several other cytokines clustered in this location [174]. It is a pro-inflammatory cytokine secreted mainly by APCs, e.g., DCs cells with chemoattractant properties towards several populations of white blood cells, including the representatives of innate and adaptive immune systems alike. The principal cell targets attracted by chemotaxis towards the CCL2 signals are monocytes, giving it the alternative designation—monocyte chemoattractant protein-1 (MCP-1) [175]. Recently, Proma et al. found that CCL2/MCP-1 was decreased in the serum of MDD patients [176]. Myung et al. reported a similar association between MDD and low CCL2/MCP-1 (further only as CCL2) levels. It responded well to antidepressant treatment, rising to similar levels found in healthy controls. The authors hypothesized that CCL2 might act as a neuroprotective chemokine, positively affecting the central dopaminergic pathways [177]. Moreover, Janelidze et al., found that CCL2 levels in the plasma and CSF were significantly decreased in suicide attempters [178]. On the other hand, various contradictory results have been published correlating the increased serum levels of CCL2 with MDD development. For example, Simon et al., examined the serum level of 20 cytokines in total, and CCL2 was among the many elevated in MDD patients [179]. Although using a different methodology, Zhou et al., found a similar association between high plasma levels of CCL2 and the development of early pregnancy depression. The authors correlated its plasma levels with the levels of LPS as a marker of microbial translocation (the occurrence of gut microorganisms in extraintestinal locations) [180]. These conflicting reports indicate that the roles of CCL2 are pleiotropic, so the definitive interpretation of its central and peripheral levels in MDD patients has to be backed up by a more robust body of data. As reviewed by Curzytek and Leśkiewicz, the chemotactic activity of the CCL2–CCR2 axis (receptor–ligand axis) is only a part of the picture. It might as likely contribute to neurodegeneration and neuroinflammation as to neuroregeneration and neurotransmission [181].

### 2.16. CCL3

Macrophage inflammatory protein-1 alpha (MIP-1α or CCL3) is monokine encoded by the *CCL3* gene, located at 17q12. It plays a crucial role during acute inflammation and is responsible for the recruitment and activation of polymorphonuclear leukocytes [182]. Hoge et al., showed that increased levels of MIP-1α with other pro-inflammatory cytokines are associated with panic disorder, post-traumatic stress disorder, and depression [183]. Merendino et al., demonstrated overexpressed MIP-1α in combination with fractalkine in patients with moderate to severe depression [184]. Depression with anxious distress is a clinically relevant subtype of MDD associated with higher levels of lipopolysaccharide-stimulated inflammatory markers, including MIP-1α [185]. Another study revealed a correlation between daytime melatonin with MIP-1α, CCL2, and VEGF-A in young patients with anxiety disorder [186]. A more recent study demonstrated that elevated serum levels of MIP-1α and iNOS are associated with post-stroke depression [187]. Moreover, increased levels of MIP-1α and other chemokines correlated with anxiety and depression during the late stages of pregnancy [188].

### 2.17. CCL5

The chemokine CCL5 (RANTES) is a chemokine encoded by the *CCL5* gene with a cytogenetic location of 17q12. Data on CCL5 are scarce, as studies on this individual chemokine are only lately being conducted. CCL5, CCL2, and CCL11 were significantly associated with anxiety and depression in a study on the uterine–chemokine–brain axis [189]. A study on job stress outcomes reported lower serum CCL5 to correlate with higher anxiety scores [190]. Period Circadian Regulator 2 (Per2)-deficient mice were immune to centrally administered LPS upregulation of CCL5 and thus less likely to suffer from neuroinflammation-induced depression-like behavior [191]. A study on pregnant women in the third trimester correlated CCL5 and other chemokines with higher anxiety and anxiety/depression prevalence. However, after adjustment for clinical measures, the association with CCL5 did not prove significant [188]. A study from Polish authors reported higher CCL5 levels in subjects compared to controls, while the levels of IL-1β and IL-6 were significantly higher in subjects [192]. A meta-analysis of 73 studies did not find any significance in blood CCL5, while lower CCL4 and higher CCL-2,3,11 with CXCL7 and IL-8 were associated with depression [193].

### 2.18. CCL11

The *CCL11* gene codes CCL11, also known as Eotaxin-1, with a cytogenetic location of 17q12. CCL11 is produced by T and B cells, endothelial cells, microglia, astrocytes, macrophages, eosinophils, and other cells in reaction to interaction with IL-4, IL-10, and IL-13. CCL11 functions also include a powerful attraction of eosinophils, basophils, neutrophils, macrophages, Th2 cells, and mastocyte cell precursors. In humans, its levels increase with age, and CCL11 is associated with cognitive and memory impairments [194,195,196]. In mice, CCL11 was found to promote microglia migration and activation with subsequent production of reactive oxygen species, potentiating glutamate-induced neuronal death [196]. In agreement with these findings, CCL11 levels in patients with bipolar disorder were found to negatively correlate with the left superior temporal volume, pointing to potential CLL-11 involvement in the pathophysiology of the disease via accelerated brain aging, and elevated CCL11 levels were also observed in MDD, regardless of the treatment [197,198]. Grassi-Oliveira et al. compared chemokine profiles in female patients with MDD with and without suicidal ideations and healthy controls. Although in their work, CCL11 was not elevated in suicidal compared to non-suicidal MDD patients, their findings confirm elevated CCL11 levels in MDD subjects compared to healthy controls [199]. However, later research comparing cytokine and chemokine profiles in depression and dysthymia reported elevated CCL11 in connection with other cytokines creating a specific network architecture in dysthymia. Still, in this particular research, CCL11 levels were similar to those of healthy controls [200].

### 2.19. Tumor Necrosis Factor

Tumor necrosis factor (TNF) is a multifunctional pro-inflammatory cytokine of the TNF superfamily encoded by the *TNF* gene located at 6p21.33 [201]. First introduced as TNF due to its capability of inducing necrosis in various neoplasms in mice, it was later renamed TNF-α because a previously described factor known as lymphotoxin was found to be homologous to TNF. Thus, its name was changed to TNF-β. However, the later discovery of a similar factor and its designation as lymphotoxin-β forced the researchers to change the name of TNF-β (former lymphotoxin) to lymphotoxin-α. Thus, the term TNF-β ceased to exist, and TNF-α became a meaningless “orphan term”. Therefore, in 1998, the official term of this cytokine was changed to its original TNF [202]. Despite that, a great bulk of studies out there still use the term TNF-α today. We will use the official term TNF in all the studies cited in this section, disregarding the fact that most use outdated terminology.

A recent paper by Benedetti et al. evaluated the examination of peripheral levels of several pro-inflammatory cytokines, including the TNF, as a predictive method to assess the success of antidepressant therapy. TNF elevation at the baseline was associated with worse treatment outcomes [203]. Das et al., evaluated the relationship between TNF serum levels and MDD. They found that TNF was not only increased in MDD, but the levels were also directly proportional to its severity. Therefore, the peripheral levels of TNF might have a predictive value in clinical practice [204]. In 2020, Bialek et al., were the first to study the SNPs in various cytokine-coding genes, including the *TNF* gene, namely c.-1211T > C—*TNF-α* (rs1799964) and c.-488G > A—*TNF-α* (rs1800629) and its relation to MDD development and treatment effectiveness. The preliminary results showed that the C allele in the C/T genotype of rs179964 was associated with positive treatment outcomes and low serum levels of TNF. These results indicate that the molecular biological approach can provide additional information beneficial for a complex diagnostic assessment of MDD and its treatment prediction [205]. Ng et al., published a systematic review and meta-analysis on the relationship between peripheral levels of TNF and four other cytokines in the elderly diagnosed with depression and AD. The primary conclusion was that there was no difference in the TNF levels between study groups and controls in either of the reviewed disease entities [38]. On the contrary, a meta-analysis by Dowlati et al. found the opposite. Those studies that met the inclusion criteria showed a significant rise in TNF serum levels compared to controls [165]. All in all, peripheral TNF levels seem to reliably correlate with MDD development, its severity, and its response to treatment. However, whether the elevation of this pro-inflammatory cytokine is in a direct causal relationship with MDD and other psychiatric disorders is still obscure. It can also be the case of reverse causality or residual confounding. Such ambiguity applies to most of the discussed cytokines. These questions were addressed in a bi-directional two-sample Mendelian randomization study by Perry et al. Still, as far as TNF is concerned, the authors did not obtain any conclusive results [206].

### 2.20. Soluble TNF Receptor 2

Soluble TNF receptor 2 (sTNFR2) is a cleaved extracellular domain of the TNFR2, also known as tumor necrosis factor receptor superfamily member 1B (TNFRSF1B) encoded by the *TNFRSF1B* gene located at 1p36.22 [207]. The cleavage of sTNFR2 is carried out by the enzyme TACE, releasing it into circulation [208]. In 2017, Bobińska et al. conducted a genetic analysis of TNFR2 along with TNFR1 and TNF to investigate whether their increased expression correlates with recurrent depressive disorder (rDD). The n = 89 study group displayed a significantly higher expression of all the genes, and their expression was also found to be inversely proportional to the performance of the patient’s cognitive faculties, including learning effectiveness, working memory, attention, and others [209]. Yamamori et al., chose to address the limitations of standard diagnostic tests for mood disorders, including MDD, by evaluating the diagnostic utility of the multi-assay biological diagnostic test. The test combined the examination of plasma levels of sTNFR2 along with two other markers. The correct classification rate of the diagnosis based on the elevated sTNFR2 plasma levels and the other two markers was 69.2%, with 62.5% sensitivity and 82.5% specificity, suggesting the potential future diagnostic value of such an approach [210]. Papakostas et al. opted for a similar study design. Their multi-assay serum-based biological diagnostic test comprised nine markers, one of which was sTNFR2. The examination of this panel of markers, which included the elevated sTNFR2 levels, was 91.1% sensitive and 81.3% specific in correctly diagnosing MDD in the test subjects. Unsurprisingly, broadening the assay and appropriate marker selection has more significant benefits compared to a narrower panel. Still, overall, the diagnostic potential of muti-assay biological tests is high, addressing the downsides of standard tests [211]. Grassi-Oliveira et al. and Diniz et al. also reported increased serum/plasma levels of sTNFR2 in MDD patients and their potential value in clinical practice [212,213].

On the other hand, Schmidt et al. found sTNFR2 serum levels to be decreased in MDD. Based on these results, the authors hypothesized about the potential explanation of such a finding. The authors’ main interpretative avenue was that the sTNFR2 is essential in maintaining a normal balance of T regulatory (Treg) and T effector cells. Therefore, the absence or subpar activity of sTNFR2 may lead to T-cell differentiation modes associated with MDD. Another explanation might be that sTNFR2 has a vital role in the normal activity of several cortical regions associated with emotional responses such as the anterior cingulate cortex, dorsolateral prefrontal cortex, and hippocampus, so its under-expression might contribute to MDD pathogenesis [214]. This vital role of TNFR2 in normal brain function was also evidenced by Pillai et al., who measured CSF levels of sTNFR2 and evaluated two SNPs in the *TNFRSF1B* gene in patients with AD. The authors found that CSF levels of sTNFR2 and the specific *TNFRSF1B* gene variations can serve as markers of resilience to cognitive impairment associated with the disease [215]. Taken together, sTNFR2 seems to be yet another valuable analyte with the potential to enrich the modern diagnostic assessment of MDD patients, provided that its measurement is appropriately combined with other biomarkers.

### 2.21. Transforming Growth Factor-Beta

Transforming growth factor-beta (TGF-β) is a multifunctional cytokine that belongs to the TGF-β superfamily of soluble, dimeric peptides. Their primary function is to control and initiate the differentiation and proliferation of various types of cells. The *TGFB1* gene encodes TGF-β with a cytogenetic location of 19q13.2. Signaling via the TGF-β/Smad pathway is essential in regulating stress response and has also been implicated in developing mood disorders [216]. TGF-β signaling has been researched concerning prenatal maternal depression and children’s brain development in utero [217]. Interestingly, lower gene expression of TGF-β was linked to larger amygdala volumes in these children. Deficits in signaling are not only common in patients with depression but also in AD [218]. In AD patients, targeting the TGF-β pathway could be a novel therapeutic approach [219]. In the inflammatory pathway, TGF-β acts as an anti-inflammatory factor, often countering the effects of IL-6. Their imbalance, combined with an imbalance in Th17/Treg cells, leads to chronic stress-induced depression in mice [220]. Moreover, gene polymorphisms in the *TGFB* gene (TT, +869) lead to lower TGF-β1 expression has been associated with impaired immunosuppression by Th3 cells and leading to depression [221]. A gene–environment (GxE) interaction study showed significant TGF-β and A2M (Alpha-2-Macroglobulin) interactions with emotional, physical, and sexual abuse [222]. Chronic fatigue syndrome in adolescents has been researched in a clinical trial that observed no significant differences in plasma samples, while TGF-β isoforms showed a relation to fatigue score [223]. All attributes mentioned above of TGF-β lead to the conclusion of a significant relation in the inflammatory depression pathway.

## 3. Conclusions

Mental disorders are a variable group of diagnoses with variable presentations, of which depression is the most prevalent. As described, it lowers patients’ quality of life, which medical professionals strive to improve, yet it often resists treatment, and quantification of treatment effectivity is impossible. Pro-inflammatory and anti-inflammatory cytokines, their receptors, and ligands play an essential role in the development and persistence of depressive symptoms [224]. A decrease in these inflammatory processes is beneficial to patients with depression, be it through medication, psychotherapy, or mindfulness training. They can reduce risk factors and prevent onset and relapses [225,226]. Multiple cytokines exhibit the potential to describe various aspects of depression and its further management. Markers of severity or resilience, diagnostic markers, markers of therapeutic effectivity, or prognosis are only some of their vast potential uses in the future. Examining cytokines with neuropeptides and finding their correlations and mutual influence could have a beneficial effect.

This review has looked closely at inflammation and associated cytokines that potentiate or react to depression. We can conclude that cytokines such as IL-1β, IL-6, IL-12, and IL-18 with IFN-γ and TNF-α are only some of the viable options for researchers to establish a quantitative method, preferably using bodily fluids with a non-invasive possibility of collection.

In conclusion, our findings indicate that cytokines are a viable option for depression research in diagnosis and treatment, as this immense burden needs to be addressed and management of this disease effectively quantified and measured.

## Figures and Tables

**Table 1:** Cytokine groups with participating receptors and ligands.

Group	Cytokines	Soluble Receptors
Interleukins	IL-1β IL-3 IL-4 IL-5 IL-6 IL-9 IL-10 IL-12 IL-13 IL-17A IL-18	IL-1 receptor antagonist Soluble IL-2 receptor
Interferons	IFN-γ	
Chemokines	IL-8 CCL2 CCL3 (MIP-1α) CCL5 (RANTES) CCL11 (Eotaxin-1)	
Lymphokines	IL-4 IL-6 IFN-γ	Soluble IL-2 receptor
Tumor necrosis factor superfamily	TNF	Soluble TNF receptor 2
Transforming growth factor superfamily	TGF-β	

## Data Availability

Not applicable.
